# Spatiotemporal dynamics of northern Caspian shorelines (1985–2023) and implications for coastal management: Lessons from the Aral Sea

**DOI:** 10.1371/journal.pone.0325546

**Published:** 2025-06-04

**Authors:** Zihao Duan, Guangsheng Wang, Jielong Hu, Tong Yu, Songgui Chen, Yajing Zhang, Yang Wang, Haicheng Liu, Xu Zhao, Hanbao Chen

**Affiliations:** 1 National Engineering Laboratory for Port Hydraulic Construction Technology, Tianjin Research Institute for Water Transport Engineering, Tianjin, China; 2 Key Laboratory of Ecosystem Network Observation and Modeling, Institute of Geographic Sciences and Natural Resources Research, Chinese Academy of Sciences, Beijing, China; 3 China Harbor Engineering Company Limited, Beijing, China; Maria Curie-Sklodowska University: Uniwersytet Marii Curie-Sklodowskiej, POLAND

## Abstract

Dynamic changes to the northern Caspian Sea shoreline have significant ecological implications, including impacts to biodiversity and the surrounding environment. This study employs Landsat datasets, historical records, and geographic information systems (GIS) to quantitatively analyze spatiotemporal variations along the northern Caspian Sea coastline from 1985 to 2023. The findings demonstrate pronounced cyclic variations in the Caspian Sea’s water level. Compared to 1930, the water level decreased by 2.6 m by 2023, with 1935 marking the onset of a significant downward trend. From 1995 to 2023, a pronounced decline in the water level at a rate of 6.1 cm/year was observed. Multiscale temporal oscillations in water levels revealed periodic rises and falls with cycles ranging from 6–8 years to 10–16 years. Due to the broad and shallow morphology of the northern Caspian Sea, fluctuations in water level have resulted in significant displacements of the northern coastline. Between 1985 and 2023, the shoreline length decreased by 262 km, which is equivalent to a 17% reduction. The intensity of the coastline length index reached a critical point during from 2010 to 2015, after which it declined sharply by 3.67. By 2023, the coastline had shifted seaward by 1.33 × 10⁴ km^2^ relative to that in 1985. This continuous retreat of the shoreline poses a severe threat to the ecological stability of the northern Caspian Sea. If the trend persists, then the disappearance of the eastern basin of the South Aral Sea may be replicated in the northern Caspian Sea by 2100. These findings provide critical insights for formulating effective coastal management strategies and conservation initiatives.

## 1 Introduction

Between 1992 and 2020, 53% of global water bodies experienced a significant reduction in storage because of global warming and increasing water demand [1]. Worldwide, closed basin seas, such as the Aral Sea, Dead Sea, Great Salt Lake, Lake Chad, and Lake Urmia, face severe hydrological decline due to human activities and climatic pressures. The near-disappearance of Aral Sea stands as a stark example of anthropogenic-induced environmental collapse. Similarly, the Dead Sea has dropped 45 m over 50 years under the effects of water diversion from the Jordan River and intensive mineral extraction [2]. The decrease in Lake Chad’s surface area by over 90% since the 1970s caused by exacerbated by climate shifts and rising water demand has devastated local communities through desertification and livelihood loss [3]. Moreover, Lake Urmia lost 60% of its area between 1995 and 2013 due to agricultural water extraction and prolonged drought [4]. These cases underscore the vulnerability of closed basins to unsustainable water management and climatic stressors and the threats to ecosystems and human populations alike [5].

The Caspian Sea (CS), situated at the intersection of Asia and Europe, represents the largest enclosed inland water body on Earth [6]. In recent years, the northern region of the CS has undergone substantial shoreline transformations due to the intricate interplay between natural processes and human-induced activities [7]. Achieving a comprehensive understanding of these shoreline changes is critical for the effective management of coastal resources and the accurate prediction of future trends under diverse climate scenarios [8,9].

Remote sensing technology represents a broad and efficient method of collecting landscape information, and it has significantly advanced the study of coastline dynamics [10]. By leveraging satellite and aerial imagery, remote sensing enables the monitoring of extensive geographical regions and the detection of temporal changes with high spatial resolution [11]. This capability facilitates the analysis of large-scale trends in shoreline movement, sedimentation patterns, and land cover changes, which are challenging to achieve through traditional ground surveys. Numerous studies have successfully employed remote sensing to investigate shoreline changes [12–15]. For instance, Kuleli et al. [12] analyzed shoreline change rates in the Ramsar wetlands of Turkey and identified significant alterations in the Yumurtalik and Goksu regions using Landsat imagery. Wang et al. [15] applied the Digital Shoreline Analysis System (DSAS) model to assess coastline changes in Tianjin, China, and used Landsat data to quantify the spatiotemporal distribution of the coastline between 1985 and 2020. These studies highlight the critical role of remote sensing in monitoring coastal dynamics and provided essential insights into variations in coastline length and fractal dimensions across diverse regions.

As a closed basin, the CS exhibits high sensitivity to climatic variations and anthropogenic activities [16]. Mapping of dynamic changes along the southern CS coastline revealed yearly fluctuations and considerable periodical changes [17]. An analysis of Landsat imagery revealed that the shoreline of the extreme southern part of the Gomishan Lagoon of the CS exhibited forward displacement into the sea of 135–7781 m [18]. Rasuly et al. [17] analyzed the southern CS coastline using Landsat imagery and object-oriented methods and revealed a 2.6 m rise in sea level (1984–1995) that caused a loss of 185 km^2^ coastland, thereby severely impacting Babolsar’s agricultural/residential zones. Ataei et al. [19] enhanced the Bruun Rule with landward sediment transport and a wave-sediment coefficient to predict the shoreline retreat of the southern CS under sea-level fall from 2013 to 2015 and revealed reduced prediction errors. Toorani et al. [20] analyzed southern CS shoreline changes using satellite imagery and DSAS. Rapid sea-level rise (1977–1995) causes erosion, while sea level decline (1955–1977, 1995–2002, 2002–2018) leads to accretion. Changes in the shoreline of Tajan closely tracked the changes in sea level, while changes in the shoreline of Sefidrud occurred due to river sediment dynamics and deltaic processes. Kakroodi et al. [21] analyzed shoreline changes along the southern Iranian Caspian coast using Landsat data collected from 1977 to 2001 and revealed that rapid sea-level rise triggered landward shifts and localized seaward shifts near sediment-rich deltas, thus highlighting the effects of sediment supply and anthropogenic factors on coastal dynamics. Akbari et al. [22] analyzed shoreline dynamics along the western CS (Astara to Chamkhaleh) and Anzali Lagoon using Landsat and altimetry data. The results revealed spatially balanced erosion (−19.22 m/year) and accretion (20.37 m/year), with shoreline movement strongly linked to sea-level fluctuations. However, deltaic sedimentation weakened this relationship in the Sefid Rud Delta.

Historical records have documented substantial water level fluctuations in the CS over centuries that were marked by distinct periods of increase and decline [23]. These variations were primarily driven by changes in the regional hydrological balance, which includes inflows, predominantly from the Volga River, as well as evaporation and precipitation variations [22]. The Volga River, which is responsible for approximately 80% of the CS’s freshwater inflow, has experienced a decrease in water level in recent years. The shallow northern region of the CS is particularly vulnerable because even minor changes in water level can result in significant shifts in the shoreline. Several studies have investigated the hydrological dynamics of the CS and their associated impacts [7,23] and revealed that the increasing rate of evaporation is the primary driver of the declining water level [24]. In addition, the crucial role of the Volga River as the primary source of inflow and the effect of climate change on the hydrological balance were emphasized [6]. The northern CS, defined by its shallow waters and expansive low-lying deltaic plains, is highly susceptible to hydrological fluctuations. Slight changes in water level can lead to significant alterations in the coastal structure, with the potential to expose large areas of seabed. A reduction in water level by 1.1 m between 2006 and 2023 resulted in a notable recession of the shoreline, with some areas of the northern coastline retreating horizontally by up to 10 m. The northern wetlands support migratory birds, Caspian seals, and fish reproduction, particularly endangered sturgeon species [24]. However, the coastal shrinkage has led to wetland loss and ecosystem service reductions [6]. Moreover, despite the significant ecological and economic importance of this region, it faces substantial risks from ongoing shoreline changes [17,25,26]. Achieving a thorough understanding of the effects of fluctuating water levels on coastal morphology is essential for informing conservation efforts, enhancing coastal management strategies, and guiding regional development plans. Nonetheless, few previous studies have focused on shoreline changes in the CS. In addition, the relationship between water level changes and shoreline transformations remains underexplored. Thus, a comprehensive long-term analysis of water level variations remains essential.

This study addresses these gaps by employing remote sensing and geographic information system (GIS) tools to quantify long-term spatiotemporal shoreline changes in the northern CS from 1985 to 2023. It aims to achieve two primary objectives: (1) identify cyclical patterns and long-term trends in water level fluctuations and (2) assess the spatiotemporal transformations in the shoreline and their ecological and socio-economic implications. The findings are expected to provide a better understanding of coastal dynamics in this vulnerable region and offer critical insights for the development of effective coastal management strategies.

## 2 Materials and methods

### 2.1 Study area

**Fig 1:**
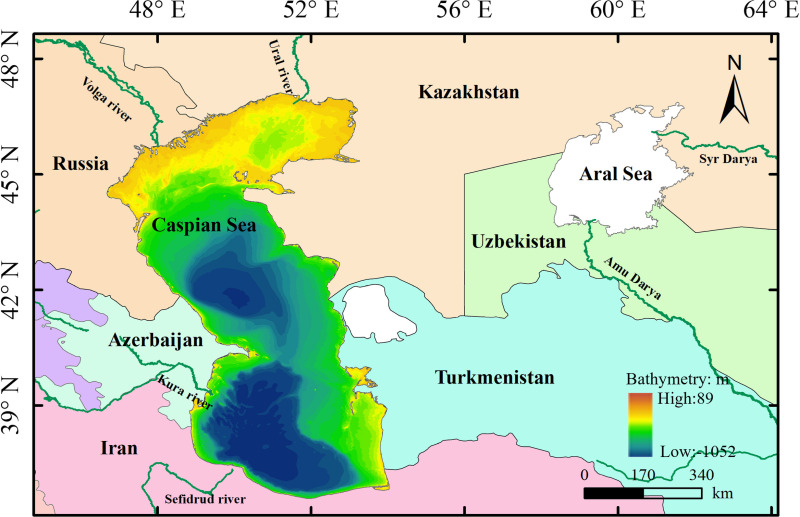
Study area of the Caspian Sea. **The map data were generated using Natural Earth** (http://www.naturalearthdata.com/).

The CS is situated to the west of the Aral Sea at the intersection of Europe and Asia and lies between Central Asia, Transcaucasia, and Iran. It has a coastline of approximately 7,000 km and a surface area of 3.7 × 10^5^ km^2^, as illustrated in Fig 1. The CS is a brackish sea with an average salinity of 13 g/L [27]. Precipitation along the coast of the central CS is unevenly distributed, with the southwestern coast receiving the highest annual precipitation (1700 mm) [28] and the northern and eastern shores experiencing significantly lower precipitation (between 100 mm and 200 mm) [6,29]. The CS represents the largest enclosed inland water body on Earth, with an average depth of 211 m. The northern region features a flat, low-lying area with depths ranging between 5 m and 10 m [26], and it contributes to the unique sensitivity of the CS to water level fluctuations. This area is ecologically significant and hosts critical habitats for species such as Caspian seals and various migratory birds [30]. The hydrological dynamics of the CS are heavily influenced by inflows from more than 130 rivers, including the Volga River, which alone accounts for approximately 80% of the total water entering the CS [31]. This inflow, coupled with the semi-arid climate in this region, leads to considerable seasonal and interannual variability in water levels, which significantly impact shoreline dynamics [22].

Given its shallow bathymetric profile and the strong correlation between water level fluctuations and shoreline displacement, the northern CS is particularly vulnerable to changes in water balance. This area is of great importance for understanding the impacts of hydrological variations on coastal morphology. Water level fluctuations can lead to significant changes in shoreline position, which has implications for both ecological stability and human activity. As such, the northern CS was selected as the study area to investigate these processes, with a focus on shoreline dynamics in response to hydrological changes. The specific characteristics of the region, including its shallow bathymetry, deltas, and estuarine systems, are critical factors that shaped the study’s methodological approach, particularly the use of remote sensing and GIS technologies to monitor large-scale shoreline changes and predict future trends [32].

### 2.2 Data source

#### 2.2.1 Hydrological data.

The water level dataset for the period 1840–1992 was compiled from previous studies [6,33,34]. While these historical datasets offer valuable insight into long-term water level trends, gaps and inconsistencies may exist due to differences in methodologies, measurement techniques, and data availability across different periods. The dataset covering the period 1992–2023 was obtained from the International Production Assessment Division (https://ipad.fas.usda.gov/), and the temporal resolution is 10 days. Data on the annual inflow from the Volga River were acquired from the Coordinating Committee on Hydrometeorology and Pollution Monitoring of the CS (http://www.caspcom.com/). Topographic information for the CS was sourced from the General Bathymetric Chart of the Oceans (https://www.gebco.net/). This bathymetric dataset offers a comprehensive view of the CS’s topography, which lacks fine spatial resolution, thereby limiting the ability to capture small-scale seabed features that may influence shoreline dynamics.

#### 2.2.2 Landsat data.

This study utilizes extensive observational datasets from Landsat 4, 5, and 7–9 to analyze dynamic changes along the northern coastline of the CS. Shoreline data were extracted from Landsat imagery covered the period from 1985 to 2023 with temporal and spatial resolutions of 16 days and 30 m, respectively [35]. These valuable datasets were systematically organized and processed using the Google Earth Engine platform (https://earthengine.google.com/) [36], which enabled large-scale spatial analysis and efficient time-series processing. To accurately delineate the coastline, the Modified Normalized Difference Water Index (MNDWI) was applied. A detailed explanation of this methodology is provided in the following section.

### 2.3 Methodology

#### 2.3.1 Mann–Kendall trend analysis.

The long-term trend of water levels on an annual timescale was estimated using Sen’s slope, a non-parametric method that is widely used for trend analysis due to its robustness against outliers and its suitability for non-linear trends [37]. To assess the statistical significance of the water level trends, the non-parametric Mann–Kendall (MK) trend test was applied, with statistical significance determined at the 95% confidence level [38]. The MK test revealed statistically significant declining or increasing trends in the water level. This method was selected due to its ability to detect monotonic trends in time series data without assuming a specific functional form, making it suitable for hydrological data where trends may be irregular or non-linear. It is worth noting that temporal autocorrelation was not considered. In addition, the Pettitt test was employed to detect the point of abrupt change in the water level trend [35].

#### 2.3.2 Continuous wavelet analysis.

The Continuous Wavelet Transform (CWT) provides an effective method of identifying the temporal and frequency characteristics of non-stationary trends [39]. The CWT allows for the decomposition of non-stationary time series into both time and frequency domains, thus providing a powerful tool for identifying periodicities in long-term hydrological fluctuations, which may vary over time [40,41]. While the CWT offers detailed insights into temporal and frequency changes, it does not directly address the underlying causes of these fluctuations. Thus, further analysis should be carried out coupled with the collection of additional data. In this study, the CWT was employed to determine the periodicity of long-term water level fluctuations.

#### 2.3.3 Coastline extraction.

In remote sensing, the reflectance characteristics of various ground objects result in varying luminance levels. To emphasize water-related features, the Normalized Difference Water Index (NDWI) has been calculated based on specific spectral bands [42]. However, the NDWI tends to overlook buildings and soil, leading to higher reflectance in the green band compared to the near-infrared band. Consequently, features extracted using the NDWI can be erroneously classified as soil or building elements, resulting in inaccuracies. To address these limitations, Xu [43] proposed the MNDWI, which utilizes the ratio of short-wave infrared to green bands [15]. This approach improves the distinction between water bodies and man-made structures, thereby addressing shadow effects and enabling more accurate water body delineation. While the MNDWI is effective in many coastal regions, it is important to note that high salinity or pollution levels can affect the accuracy of the method.

**Fig 2:**
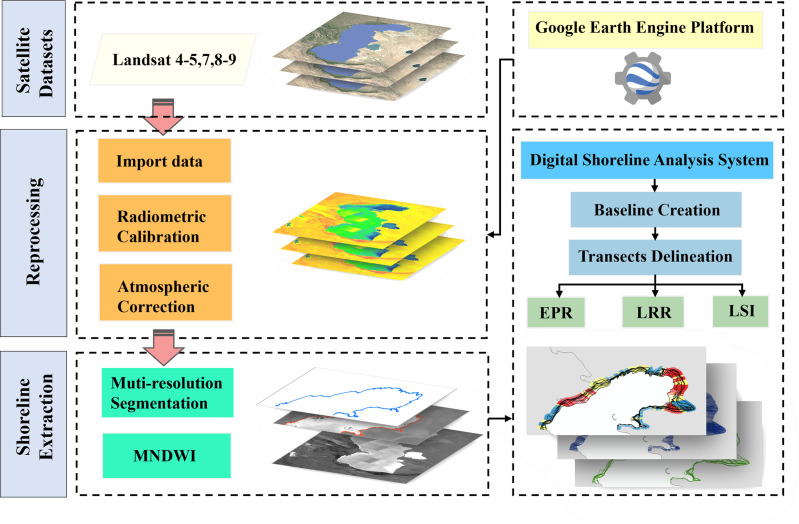
Methods used to extract shoreline changes.

The MNDWI has been widely used for land-sea boundary delineation [44]. Therefore, the MNDWI was applied to improve the contrast between wet and dry coastal areas, which facilitated the accurate extraction of shoreline features. The formula for the MNDWI is as follows:


$$\begin{array}{c} MNDWI=\frac{Green-MIR}{Green+MIR}, \end{array}$$ (1)

where “Green” refers to the green spectral band, and “MIR” denotes the middle-infrared band. The dataset underwent a comprehensive preprocessing procedure, including image correction, enhancement of water bodies, binarization through thresholding, noise reduction, radiometric calibration, geometric rectification, cloud removal, and image mosaicking. Subsequently, coastline delineation from Landsat imagery was conducted for the period spanning from 1985 to 2023. The methods employed to extract shoreline changes are presented in Fig 2.

#### 2.3.4 Digital shoreline analysis system (DSAS) model.

The DSAS model is widely employed to estimate shoreline migration rates [45,46]. The evolution of the coastline was determined by comparing temporal coastlines with established baseline positions. The landward baseline followed the general shoreline trend. A fixed transect interval of 300 m was used, which provided a balance between computational efficiency and accuracy. However, this approach may overlook finer-scale variations, particularly in regions with complex coastal morphology (e.g., estuaries). The rate of shoreline changes for each transect was calculated using linear regression. Both the endpoint rate (EPR) and linear regression rate (LRR) methods were applied to evaluate shoreline changes [47].

#### 2.3.5 Intensity of coastline changes.

The average rate of change method was used to quantify the spatiotemporal intensity of coastline changes [15] and calculate coastline changes in the CS as follows:


$${LSI}_{mn}=\frac{\left(L_{m}-L_{n}\right)}{L_{n}(m-n)}\times 100\;\%,$$ (2)

where *LSI*_*mn*_ represents the intensity of coastline length change between years *m* and *n* (where n > m) and *L*_*m*_ and *L*_*n*_ denote the coastline lengths of the corresponding year. A higher $\left|{LSI}_{mn}\right|$ indicates a greater intensity of coastline change.

#### 2.3.6 Lake volume change.

To calculate changes in water volume, the method applied by Zhang [48] was utilized. Volume change (ΔV) was determined using topographic information coupled with the following formula:


$$\Delta V=\frac{1}{3}\left(H_{1}-H_{0}\right)\times \left(A_{1}+A_{0}+\sqrt{\left(A_{1}\times A_{0}\right)}\right),$$ (3)

where *H*_*0*_ and *A*_*0*_ represent the initial water level and area of the sea, respectively, and *H*_*1*_ and *A*_*1*_ correspond to the values during the study period. For volume calculations, the area data closest in time to each water level measurement were selected.

## 3 Results

### 3.1 Water level changes

**Fig 3:**
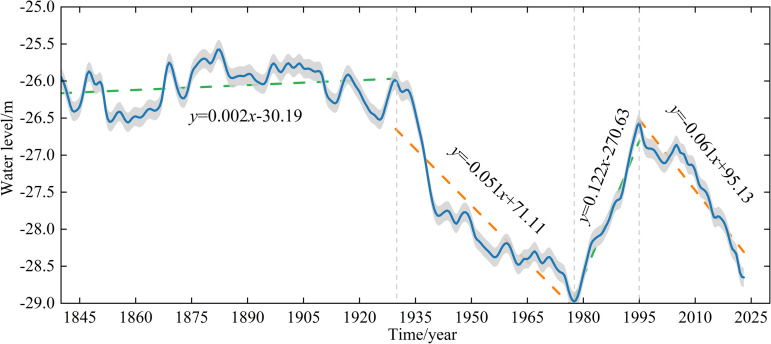
Water level changes in the Caspian Sea.

Since 1840, the CS has undergone significant changes in water levels, characterized by both long-term and short-term fluctuations. A detailed examination of water level data from 1840 to 2023 reveals a notable decline that began in 1930, with a total decrease of 9.9% by 2023 compared to the water level recorded in 1930 (Fig 3). During the period from 1840 to 1930, the water level exhibited an average annual increase of 0.2 cm/year, reaching an average elevation of −26.07 m, with a maximum variation of 0.99 m. However, between 1930 and 1977, the water level experienced a sharp decline at a rate of 0.05 m/year. This decline is primarily attributed to intensified human activities. The heavy exploitation of the Volga and Ural rivers occurred since the 1960s, including the construction of dams and increased water withdrawals for agricultural irrigation, which significantly reduced freshwater inflows into the CS [49,50]. Although subsurface flow into the sea, previous studies have shown that the contribution of subsurface to water level fluctuations is small, and groundwater inflow is negligible compared to riverine inflow [25,31]. The resultant reduction in water levels (approximately 1 m) and decrease in the surface area (2.2 × 10⁴ km^2^) were notably more pronounced in the northern region [27].

**Fig 4:**
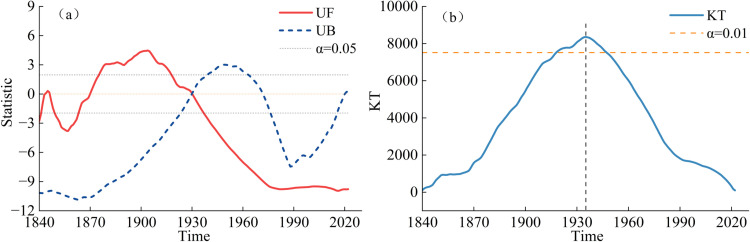
(a) M-K test of annual average water level in Caspian Sea (unformatted fit (UF) and underidentification bias (UB) is positive and reverse order in the time series respectively; ɑ is the significance level); (b) Pettitt test statistic (ɑ is the significance level, KT is the statistic of Pettitt test).

Caspian Sea level (CSL) variability is influenced by the complex interaction between climatic oscillations and regional hydroclimatic feedback. While ENSO seasonally affects precipitation in the Volga basin, its limited correlation with CSL during the summer months underscores the importance of other drivers [51]. The North Atlantic Oscillation (NAO) and Siberian High (SH) play pivotal roles in modulating moisture transport. A positive NAO phase enhances winter westerlies, which increase precipitation in the Volga catchment, while wind anomalies associated with the SH system amplify evaporation [52,53]. CSL decline is further linked to wind-driven evaporation processes. Strengthened westerlies export moisture out of the basin, reducing precipitation recycling, while anti-cyclonic easterly winds increase localized evaporation from the eastern catchment [31]. These interactions create a negative feedback loop, where changes in CSL influence the regional evaporation-precipitation balances. Subsurface hydrological processes and decadal oceanic-atmospheric oscillations, such as Pacific Decadal Variability, remain underexplored due to computational constraints. From 1977 to 1995, the water level exhibited an increasing trend at a rate of 0.12 m/year, resulting in a total rise of 2.38 m, with the peak level reaching −26.57 m. This trend was mainly due to a combination of reduced water withdrawals for irrigation, increased precipitation in the Volga River basin, and relatively lower evaporation rates during this period [50]. Notably, an increase in sea level was suggested to be linked to the hydroclimatic effect of an El Nino-Southern Oscillation teleconnection [51]. However, this elevation did not return to the average levels observed in 1930. The increase in water level is attributed to a reduction in irrigation withdrawals, increased precipitation, and lower evaporation rates during this period. The observed correlation between coastline expansion from 1978 to 1995 and heavy precipitation events in the Volga River basin further supports this interpretation [53]. From 1995 to 2023, the water level showed a downward trend at a rate of 6.1 cm/year, which was greater than the rate observed from 1930 to 1977, with the lowest water level recorded at −28.59 m in 2023. A notable decrease in water level occurred in 2010 due to the northern summer drought [31]. The primary cause of the ongoing decline is climate change, which has intensified evaporation rates and reduced precipitation. These changes have resulted in a lower capacity for water replenishment through precipitation, and further depleted water resources in the CS. The results of the Mann-Kendall (M–K) test indicated that the average annual water level began to decrease in 1930, with the downward trend becoming statistically significant at the 0.05 level after 1937, as shown in Fig 4a. Furthermore, the Pettitt test results indicated that the water level underwent an abrupt change around 1935 at the 0.05 significance level, as shown in Fig 4b. While these trends are statistically significant, they are subject to certain limitations. Historical data, especially from earlier periods (e.g., 1840–1930), may contain measurement errors or discontinuities, which could affect the accuracy of the identified trends.

**Fig 5:**
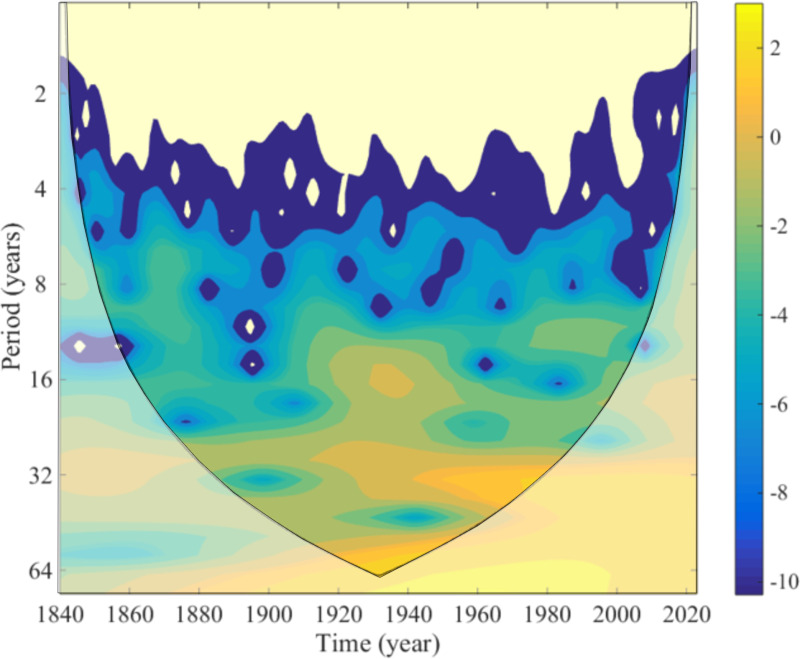
Morlet wavelet power spectrum of the sea level.

The wavelet power spectrum of the CS water level changes from 1840 to 2023 is illustrated in Fig 5. The horizontal axis represents time, while the vertical axis represents the time scale. Positive contour values indicate an increase in water levels, whereas negative values reflect a decrease. The spectrum revealed pronounced non-stationary behavior, thereby confirming the hypothesis of energy accumulation on the annual scale. High-energy signals were observed in the low-frequency range, corresponding to periods longer than 8 years. The evolution of water levels was variable across multiple time scales. The results revealed the presence of multiple oscillations on a timescale of approximately 6–8 years, quasi-triple oscillations on a timescale of 10–16 years and a single oscillation on a timescale of 30 years. Notably, the periodic change approximately every 3–5 years exhibited relatively stable behavior after the 1850s. Additionally, previous studies [54] on the KaraBogazGol system have identified longer-term cycles, with primary periodicities of approximately 62.5 years and 38.5 years. The divergence in cyclic behavior suggests that KaraBogazGol water level fluctuations are modulated by additional factors, including hypersaline dynamics, sedimentary processes, and differential evaporation patterns. The KaraBogazGol system plays a crucial role in the overall hydrological balance of the CS by acting as a terminal basin where excess CS water is lost primarily through evaporation. Unlike the CS, where water level changes are driven predominantly by river inflows (mainly from the Volga), the KaraBogazGol system is more directly influenced by localized salinity-driven evaporation cycles. These differences result in distinct long-term oscillatory behavior.

**Fig 6:**
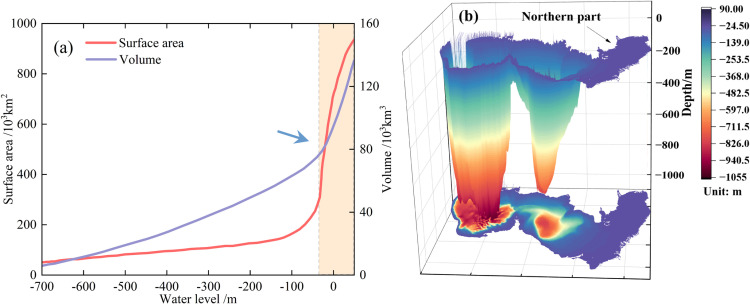
(a) Surface area and volume change with water level; (b) Topographic changes in the Caspian Sea.

The relationship between the water level, area, and water storage is presented in Fig 6a, which reveals nonlinear growth between the water area and storage. When the water level elevation was below −100 m, both the area and water storage exhibited gradual changes, indicating a “V”-shaped bathymetric profile of the seabed, as shown in Fig 6b. When the water level increased above −35 m, both the area and water storage increased significantly, which was likely related to the expansive shallow topography of the northern basin. In 2023, the water surface area was 3.95 × 10⁵ km^2^ and the volume was 7.85 × 10⁴ km^3^. This represents a reduction of 4.8% in area and 0.4% in volume compared to that in 1985, and a reduction of 11.2% in area and 1.3% in volume compared with that in 1840. Moreover, the topographic characteristics have led to distinct hydrological dynamics and shoreline transformations of the northern CS.

### 3.2 Northern Caspian Sea coastline changes

#### 3.2.1 Coastline length changes.

**Fig 7:**
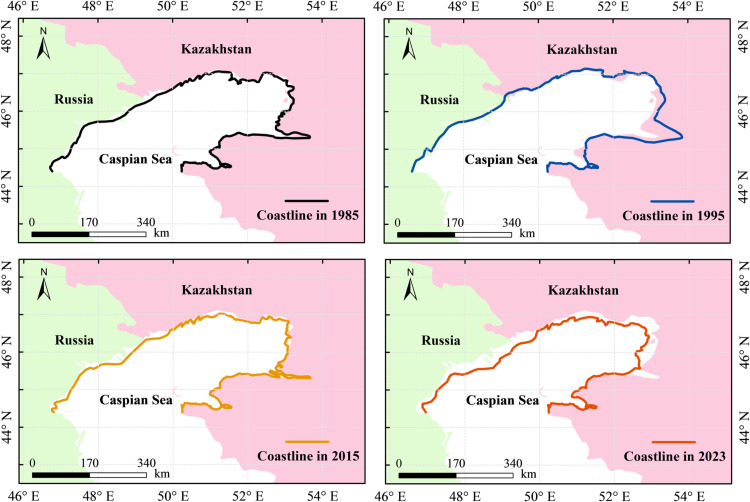
Coastline variations in different years. Map data were generated with Natural Earth (http://www.naturalearthdata.com/).

**Fig 8:**
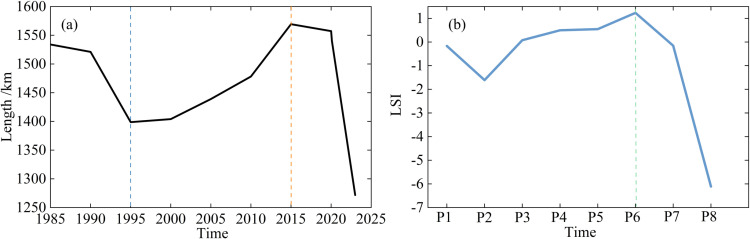
(a) Coastline length changes in the Caspian Sea; (b) LSI changes between 1985 and 2023 (P1 = 1985–1990, P2 = 1990–1995, P3 = 1995–2000, P4 = 2000–2005, P5 = 2005–2010, P6 = 2010–2015, P7 = 2015–2020, P8 = 2020–2023).

Historical records indicate that the coastline changes have involved both advances and retreats, as shown in Fig 7. Periodic rises in water levels have led to the submersion of extensive coastal plain areas, while decreases in water levels have exposed vast tracts of mudflats and wetlands. Over the past 39 years, the continental coastline has exhibited a fluctuating trend, alternating between periods of rising and falling water levels. This has ultimately resulted in a seaward migration with a net reduction in total shoreline length. The reduction in coastline length is primarily attributed to a decline in the extent of the surface area. During this period, the length of the northern coastline decreased by 262 km, representing a 17% reduction, as shown in Fig 8a. This reduction corresponds to an average annual decrease of 6.7 km. Notably, the rate of coastline changes exhibited considerable variation over time. Between 1985 and 1995, the coastline experienced a comparatively lower rate of decrease at 12.3 km/year. However, from 1995 to 2015, the coastline length increased at a rate of 8.1 km/year. Subsequently, between 2015 and 2023, the rate of decrease accelerated to 33.1 km/year. The recent acceleration in decline may be attributed to intensified evaporation, reduced river discharge, and increased sediment compaction, which have collectively influenced the stability of the northern coastline.

The LSI exhibited fluctuations across eight periods, as shown in Fig 8b. Prior to period P6, the LSI experienced a slight increase of 0.09, reaching a peak of 1.23. From P6 to P8, the LSI underwent a significant decrease, with a total reduction of 3.67. Thus, period P6 marked a critical turning point in coastline changes. During the periods 1985–1990 (P1) and 2015–2020 (P7), the progression and regression of the coastline remained approximately equal; thus the shoreline remained relatively balanced. However, in periods P2 and P8, the intensity of the regression was considerably greater than that of the progression, indicating periods of pronounced coastline retreat and extensive seabed exposure.

#### 3.2.2 Accretion and erosion.

**Fig 9:**
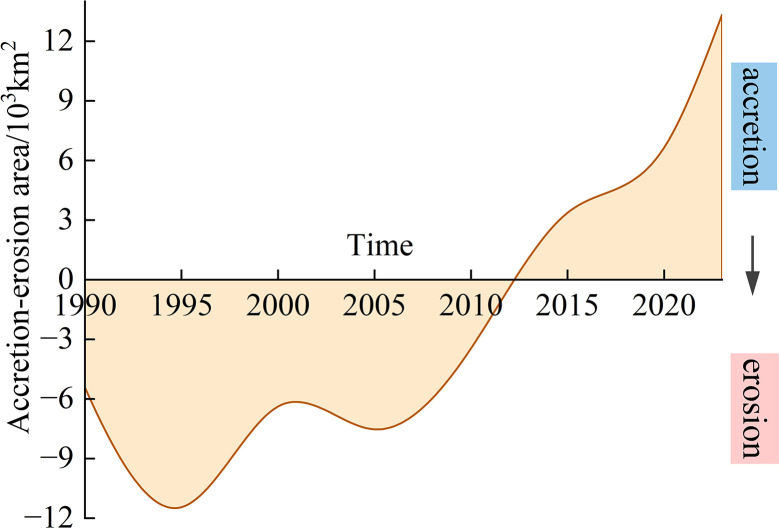
Coastal area changes during the period 1985–2023.

Variations in coastline length led to substantial changes in the spatial extent of the coastal area. Compared to 1985, the coastline advanced seaward by 1.33 × 10⁴ km^2^ in 2023 (Fig 9). The northern Caspian coastline exhibited distinct phases of both accretion and erosion, which were largely driven by hydrological and climatic variations. Between 1985 and 2012, the coastal zones were predominantly subjected to erosion, with a maximum erosion area of 1.14 × 10^4^ km^2^. Meanwhile, the sedimentation area gradually declined until 2012, reaching a level comparable to that observed in 1985. However, a notable accretion phase occurred between 2012 and 2023, with annual growth peaking at 1.36 × 10^3^ km^2^ in 2015.

The use of remote sensing datasets has facilitated the detailed observation of significant changes along the northern coastline of the CS in response to fluctuations in water levels. Analysis of satellite imagery from 2006 to 2023 revealed an increase of approximately 3.5 × 10^3^ km^2^ in the exposed seabed area in the northern CS. The northern coastline receded by 5–10 km, with the most dramatic changes occurring in the shallow deltaic regions. Specifically, the delta of the Ural River experienced substantial retreat, resulting in the exposure of over 1000 km^2^. The extensive exposure of coastal wetlands and shallow subsea areas has substantially transformed critical habitats due to shoreline retreat. The reduction in water surface area has diminished the habitat of the Caspian seal, and the loss of wetlands has reduced the populations of migratory birds and spawning habitats of fish [30]. Additionally, the decline in shallow water areas may reduce benthic productivity and negatively affect fisheries. Furthermore, the large-scale exposure of the seabed, which is subject to wind erosion, may contribute to a higher frequency of dust storms, exacerbating eco-environmental degradation in the northern CS [26].

#### 3.2.3 Change of spatiotemporal coastline.

**Fig 10:**
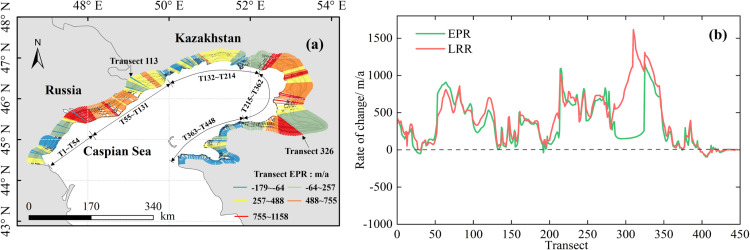
(a) Change rates were calculated for each transect along the coastlines from 1985 to 2023; (b) Coastline change rates of the Caspian Sea based on the EPR and LRR.

The coastline change rate in the northern region was assessed using the DSAS with two statistical methods: the LRR and EPR. As shown in Fig 10a, transects were generated by the DSAS, which was oriented perpendicular to baselines and intersected all shorelines. The coastal segments were categorized into different groups based on the coastline change rates derived from the EPR model. The EPR and LRR values for the various transects are presented in Fig 10b. The results revealed that 89.4% of the shorelines exhibited accretion, with the highest accretion rate of 1157.7 m/a observed in the northeastern delta area (Transect 326). High erosion rates, reaching 179.3 m/a, were predominantly observed in the area to the west of Russia (Transect 113) and Mertvy Kultuk Bay, near Kazakhstan. As shown in Fig 10a, transects 220–350 were primarily located within the northeastern estuarine delta region. Of note, the accuracy of detected shoreline changes was inherently influenced by the resolution and temporal frequency of satellite imagery as well as potential errors in georeferencing. Thus, future studies should integrate high-resolution hydrodynamic modeling and field-based validation to improve the robustness of shoreline change assessments.

## 4 Discussions

**Fig 11:**
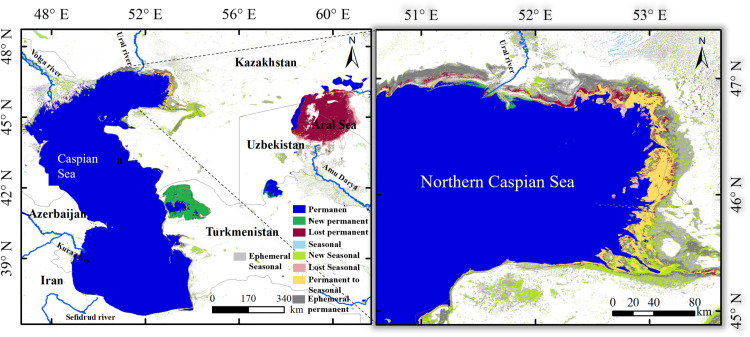
Water surface transitions from 1984 to 2021. The map data were generated by the Global Surface Water Explorer (https://global-surface-water.appspot.com/faq).

The Aral Sea and CS share many geographical and climatic similarities, as shown in Fig 11. Thus, the Aral Sea represents a potentially valuable reference for comparison when evaluating the risks facing the CS. Both are located in arid endorheic basins, heavily influenced by inflows from rivers and precipitation, and experience substantial evaporation. According to the First Law of Geography, geographical phenomena in adjacent areas typically exhibit analogous characteristics [55]. The well-documented regional features, hydrology, and ecological changes of the Aral Sea basin in Central Asia provide valuable insights that can be applied to assess the potential risks of environmental changes in the CS.

**Fig 12:**
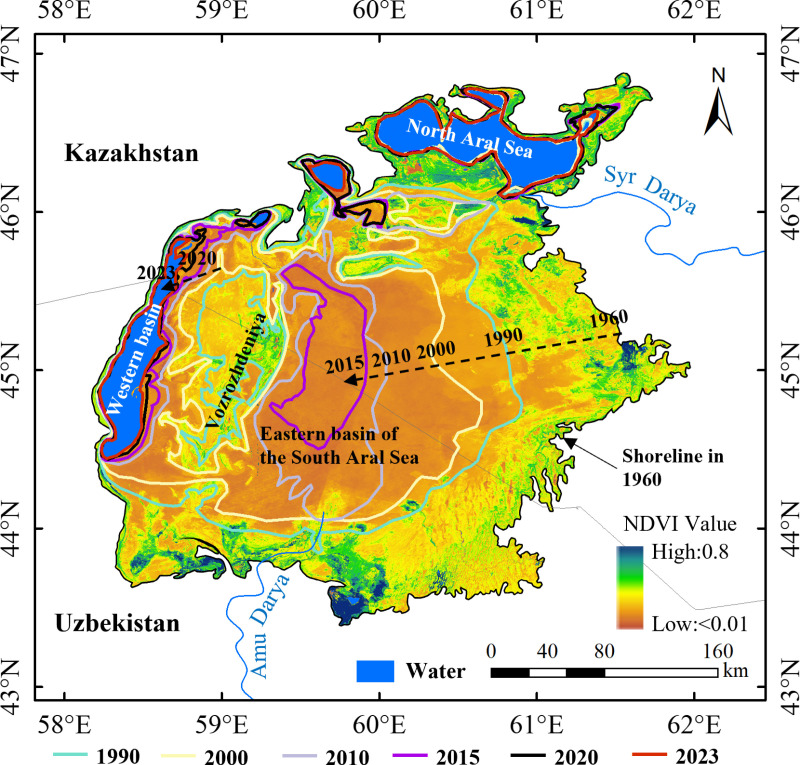
The coastline changes of the Aral Sea from 1960 to 2023. Land cover data are from LANDSAT 8 ( https://landsat.gsfc.nasa.gov/).

The Aral Sea, once the world’s fourth-largest saline sea, provides crucial insights into the severe ecological consequences of extensive anthropogenic water diversion. The dramatic shrinkage of the Aral Sea, particularly following large-scale irrigation projects that diverted water from the Syr Darya and Amu Darya rivers, presents a cautionary tale for the CS. Between 1984 and 2021, 47% of the water area was permanently transformed into land area, leaving only 8.6% as permanent water (Fig 12). By 2023, the eastern basin had disappeared entirely, leaving only a narrow strip of the western basin. The coastline along the southern Aral Sea receded by 2.73 km/year from 1960 to 2023, resulting in profound ecological degradation, including increased salinity, local fish species extinctions, desertification, and widespread toxic dust storms [56,57]. These ecological impacts have had devastating socio-economic consequences for the region, including fishing industry collapse, widespread unemployment, and public health deterioration [58–60]. The decline of the Aral Sea offers a critical lens through which to assess the potential trajectory of the CS, particularly with respect to its vulnerable ecosystems and human populations. The decline of the Aral Sea was driven by a combination of water over-extraction and regional climatic changes, which resulted in rising salinity levels and severe ecological disruptions [61].

Similarly, the CS faces analogous threats, particularly from the over-extraction of water from the Volga River. Over recent decades, the increased withdrawal of water from the Volga, compounded by rising temperatures, has accelerated the decline in CS water levels [62]. In addition, evaporation has shown an increasing trend while precipitation has shown a decreasing trend. Precipitation decreased by 90 km^3^ and evaporation increased 103 km^3^ with increases in temperature from 1992 to 2022. Climate change in the CS region has led to increased air and surface water temperatures, higher evaporation rates, and decreased CS water budgets. A significant portion of the water area transitioned initially into seasonal water occurrences and subsequently into permanent land, as illustrated in Fig 12. A significant phase of accretion occurred between 2012 and 2023, with a further 1.33 × 10^4^ km^2^ advance seaward in 2023 compared to that in 1985 (Fig 9). This accelerated decline can be attributed to the greater evaporation rates, lower river inflows, and ongoing sediment compaction. Projections suggest that by the end of the 21st century, the CS’s water level could decrease by 9–18 m, which would reduce its surface area by at least 25% and expose 9.3 × 10^4^ km^2^ of seabed [63,64]. The potential disappearance of the northern CS would result in the loss of key habitats for both marine and terrestrial species, thereby further exacerbating the decline of the Caspian seal and other important species [65]. The drying of coastal wetlands and shallow subsea areas could initiate a cascade of ecological consequences, such as shifts in species composition, the collapse of local fisheries, and the loss of ecosystem services [61]. The socio-economic challenges posed by the shrinking CS could mirror those faced by communities near the Aral Sea, including health crises exacerbated by pollution and the spread of diseases such as hepatitis and tuberculosis.

Given the similarities between the two seas, the potential for a recurrence of the Aral Sea’s ecological collapse in the CS remains significant. The continued shrinkage of the CS threatens its unique biodiversity and jeopardizes the livelihoods of surrounding communities. If water levels continue to decline at the projected rate, the loss of critical habitats, particularly the northern shallow continental shelf, could lead to the extinction of species dependent on these ecosystems, including the Caspian seal, local fish, and migratory birds. Additionally, higher evaporation rates and increasing salinity levels will likely exacerbate the already fragile environmental conditions in the CS.

**Fig 13:**
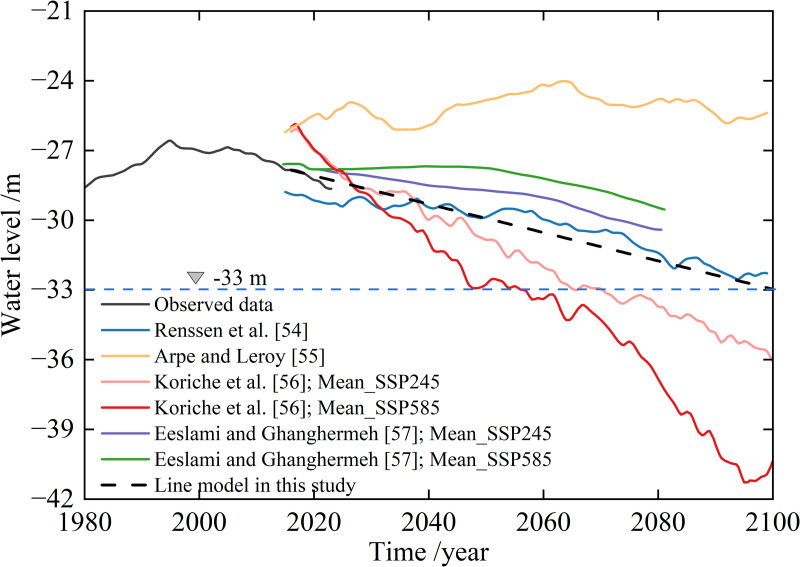
Water level changes in the foresight scenarios.

Several studies have forecast the water level of the CS, as shown in Fig 13. The water level will continue to decline in the future. Based on the observed rate of decline in water level from 1995 to 2023, the water level is projected to reach −33 m by the year 2100. If the water level drops to −33 m, then the water surface area would decrease by 0.9 × 10⁵ km^2^ compared to that observed in 2023. As a result, the northern CS would disappear, potentially leading to the extinction of the Caspian seal. Consequently, changes in the coastline will result in substantial ecological, economic, and demographic damage to coastal areas.

The CS faces severe environmental crises driven by climate change (3°C projected warming by 2100), extreme water-level fluctuations (historic decreases/increases of 4 m and 2.5 m), agricultural water extraction, and pollution from oil spills [66]. These pressures compound shoreline instability, which is exacerbated by Volga River regulation, thereby triggering erosion and habitat loss for endangered species such as sturgeon (80% population decline) and seals [67]. Biodiversity collapse intensified by the fragmented governance under the 2018 Caspian Convention, geopolitical disputes over vast oil reserves, and prioritization of short-term hydrocarbon profits over ecological sustainability. Weak enforcement of transboundary agreements, persistent disconnects between scientific data (e.g., hydrological models) and policy action, and competing economic interests have paralyzed regional management. If protective measures for the CS are not implemented, then the tragedy that befell the Aral Sea may also occur in the CS. Efficient irrigation techniques (e.g., drip irrigation, precision agriculture) should be promoted to reduce excessive water consumption in the Volga basin and international agreements should be developed among riparian states to regulate water usage and maintain ecological balance. Addressing these challenges requires resolving legal ambiguities, enforcing binding pollution controls, and integrating adaptive strategies, such as coastal zoning and data-driven planning, and aligning economic priorities with ecological resilience.

## 5 Conclusions

This investigation examined water level dynamics and coastline changes in the CS from 1840 to 2023. Additionally, a comparative analysis was performed to assess the hydrological and ecological changes under similar recessionary conditions. The main conclusions are as follows:

(1) The water level in 2023 decreased by 2.6 m, representing a 9.9% decline compared to that in 1930. The evolution process of the water level demonstrated multi-time scale characteristics. These changes, particularly the accelerated loss of water area, highlight the urgent need for effective environmental management strategies in the CS region.
(2) The DSAS analysis indicated that 89.4% of the shorelines exhibited accretion. with the most significant change occurring in the northeastern delta region. These results highlight localized variations in shoreline dynamics, which could influence coastal ecosystems and human activities, particularly in the northeastern estuarine areas. The adoption of proactive water management policies is essential, particularly those focused on the sustainable regulation of river inflows and the implementation of conservation strategies aimed at protecting vulnerable coastal ecosystems, including wetlands and migratory habitats.
(3) Based on the observed degradation in the CS, it is likely that the ecological and environmental tragedy of the eastern basin of the Aral Sea may occur in the northern CS by 2100. Limitations of this study include the reliance on historical data, which may not fully capture future hydrological trends under climate change scenarios. Future research should focus on refining predictive models and exploring the socio-economic ramifications in details.
